# Suicide by ligature strangulation and/or hanging inside a motor vehicle: a comprehensive review

**DOI:** 10.1007/s12024-024-00828-1

**Published:** 2024-05-18

**Authors:** Carlo Pietro Campobasso, Mariavictoria De Simone, Antonietta Porzio, Edoardo Mazzini, Anna Carfora, Alessandro Feola

**Affiliations:** 1 Department of Experimental Medicine, University of Campania “Luigi Vanvitelli”, via Luciano Armanni 5, Naples, 80138 Italy; 2 Department of Mental and Physical Health and Preventive Medicine, University of Campania “Luigi Vanvitelli”, via Luciano Armanni 5, Naples, 80138 Italy

**Keywords:** Asphyxia, Hanging, Vehicle-assisted suicide, Ligature strangulation, Decapitation, Forensic science

## Abstract

Suicide by ligature strangulation/hanging inside vehicles is uncommon, and only few cases have been reported in the literature. This study aimed to conduct a comprehensive review of reported cases of suicide by ligature strangulation/hanging inside vehicles, analyzing the features of the death scene, of the ligature and furrow, autopsy findings, and causes of death. The comprehensive review was performed following the PRISMA guidelines by using the most common scientific databases. According to inclusion criteria, a total of 20 cases of vehicle-assisted strangulation/hanging were reviewed: 13 cases were assessed as ligature strangulation resulting in 7 complete decapitations and 7 other cases as hanging. All victims were young or adult males, except for one 48-year-old female. Death was assessed as suicide in all cases, except for a possible accidental autoerotic death. In 8 cases, a history of depression or other psychiatric disorders was reported. Toxicological analysis were positive in 7 cases. Hard ligature materials (nylon, steel, plastic, hemp ropes) were used in most cases, but only 13 cases had a well-demarcated furrow. In 2 cases, no internal findings of asphyxia were found. An additional case of ligature strangulation inside a motor vehicle off is also presented, where no autopsy findings of asphyxia were observed, except for a broad pale furrow and monolateral conjunctival petechiae. This study highlights the challenges in classifying suicidal hanging and ligature strangulation in motor vehicles.

## Key points

1. Suicide by ligature strangulation or hanging inside motor vehicles is uncommon, with few cases documented inthe literature.

2. Determining the type of asphyxia death (ligature strangulation or hanging) for victims who died due tocompression to the neck by a ligature can be challenging.

3. Sometimes the specific subtype cannot be determined, the case should be classified as "strangulation nototherwise specified".

4. Different elements (type of ligature materials used, the presence of well-demarcated furrows, and internal signsof asphyxia) could complicate the determination of the cause of death.

5. Forensic pathologists must analyze every aspect, in order to conduct an accurate analysis.

## Introduction

Suicide is a multidimensional and challenging global public health concern. Each year, over 700,000 individuals worldwide die by suicide [1]. Suicides related to motor vehicles are not rare. The automobile can be either the location where the suicide takes place, or the tool by which it is carried out. Suicide by motor vehicles is often associated to significant blunt trauma from pedestrian hits or intentional car crashes. The victim is usually a young male suffering from psychosocial stress, mental disorders with prior suicide attempts, and a personality trait characterized by impulsivity and low distress tolerance [2]. Less traumatic mechanisms of death are usually preferred by adult males with a long history of depression or drug abuse [3]. They are mostly represented by carbon monoxide inhalation or other drug intoxication [4]. After a consistent upward trend spanning the last two decades, asphyxiation has emerged as the second most commonly employed suicide method [5]. Asphyxiation-related deaths can also occur inside a vehicle, simply as a setting where suicide is carried out. A classification of automobile-related suicides with motor vehicle as an integral part of the process has been suggested [4] and recently updated [2]. The speed and mass of the vehicle can be used to inflict lethal injuries, but also to assist drowning, carbon monoxide toxicity, self-immolation by fire, ligature strangulation, and/or hanging through the automobile fixtures (e.g., seatbelts) or ropes. A motor vehicle often represents a secure place for asphyxia deaths as it can provide the opportunity to reach an isolated area where the suicidal intent can be realized with no hurry. However, suicide caused by strangulation or hanging inside a car is rare. Few cases of hanging by using seat belts or other ropes, and vehicle-assisted ligature strangulation are reported. Aim of this study is to review the literature dealing with suicidal deaths by ligature strangulation and/or hanging inside a motor vehicle to better understand the epidemiologic characteristics of the victims, the ligature methods, and the mechanisms of death consistent with autopsy and toxicological findings.

## Materials and methods

The literature review was carried out according to the PRISMA statement [6]. PRISMA is a methodology for systematic reviews and meta-analyses, used for complete and standardized researches. Five electronic bibliographic databases PubMed, Scopus, Web of Science, Cochrane and Embase were screened. No temporal restrictions were applied. The keywords “(hanging AND car) OR (asphyxia AND car) OR (strangulation AND car) OR (hanging AND vehicle-assisted) OR (asphyxia AND vehicle-assisted) OR (strangulation AND vehicle-assisted)” were searched in all fields. The following inclusion criteria were adopted: (1) papers reporting cases of suicidal strangulation inside a motor vehicle, (2) papers reporting cases of suicidal hanging inside a motor vehicle. Exclusion criteria were the following: (1) scientific articles not published in English, (2) full text not available, (3) accidental deaths after car crashes, (4) no autopsy findings reported. For duplicate studies, only the article with more detailed information was included. Two examiners independently provided the initial selection of the articles; the title, the abstract, and the full text of each potentially pertinent study were reviewed. Disagreements on the eligibility of the studies were solved between the two examiners through a preliminary discussion between the two examiners according to the inclusion and exclusion criteria. If no agreement could be reached, it was resolved using a third co-author as reviewer. Articles were evaluated and key data extracted according to predefined criteria. The following items were recorded: victim’s characteristics, mental health problems, startup of the vehicle, position of the body, ligature, external and internal findings, toxicological results, manner of death assessed by authors.

## Results

**Fig. 1:**
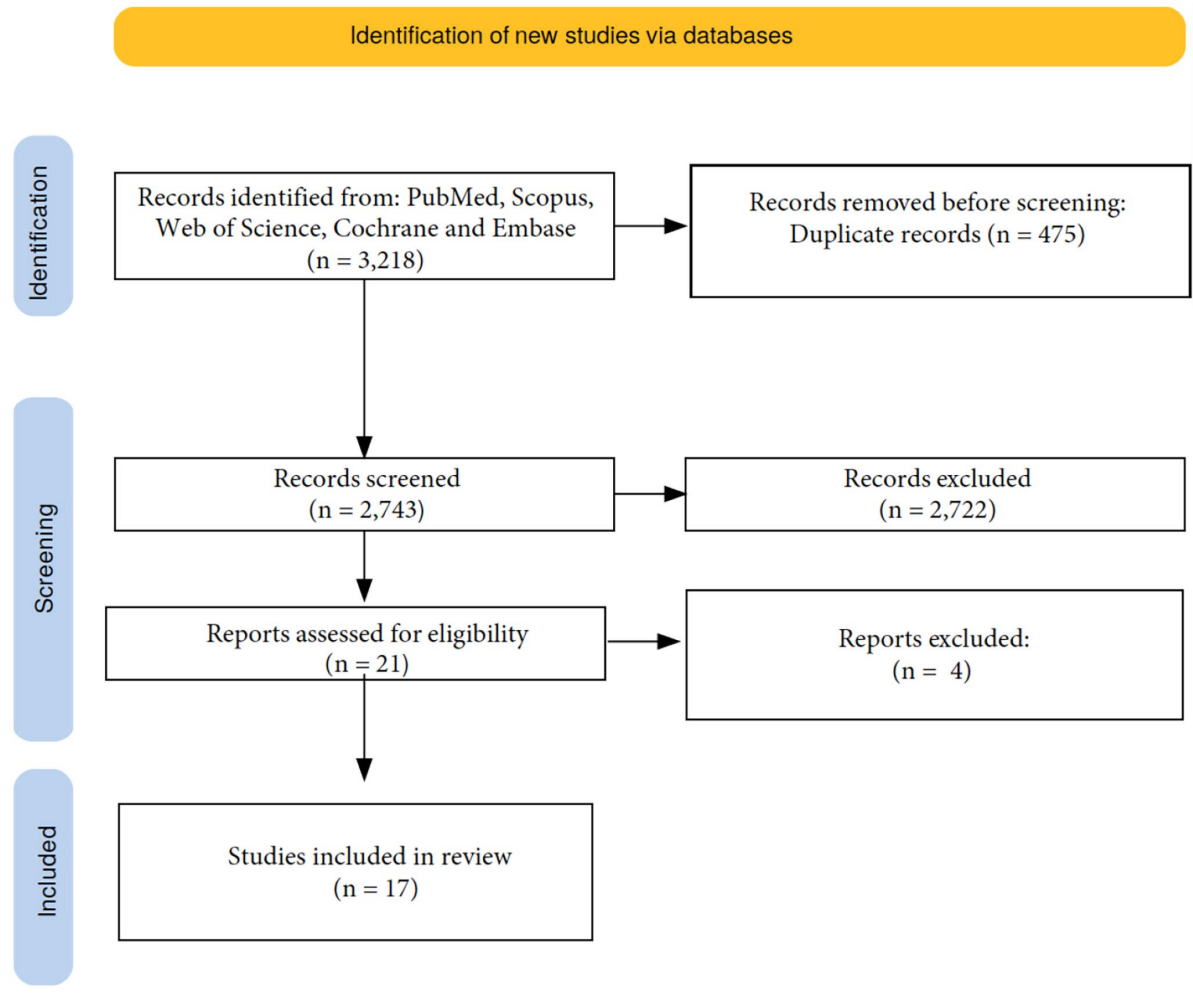
Flowchart depicting the methodology followed in the study

The search of PubMed, Scopus, Web of Science, Cochrane and Embase databases provided 3,218 articles in total: 148 from PubMed, 2,581 from Scopus, 211 from Web of Science, 71 from Cochrane and 207 from Embase. After adjusting for duplicates, 475 were discarded. After reviewing titles and abstracts, 2,726 were discarded since they did not satisfy the inclusion criteria. Finally, only 17 papers reporting 20 cases of suicidal strangulation and/or hanging inside a motor vehicle in total satisfied the inclusion criteria. A flowchart depicting the selection of studies according to PRISMA standards is reported in Fig. 1.

20 cases of suicidal strangulation and/or hanging inside a vehicle have been reported in total by 17 articles. All victims were male except for a 48-year-old female. The age of the victims ranged from 20 to 67 years. The mean age was 44.3 years. The victim’s age was not reported only in a single case report [7].

In 11 out of 20 victims, only clinical and mental health history was available, mainly represented by depression (8 cases) associated with previous suicidal attemps or notes found close to the body in the vehicle in 5 cases. In 2 cases, a history of drug and alcohol abuse was referred by relatives and confirmed by post-mortem toxicological analysis. In 4 other cases, ethanol was detected in the blood samples, among which one victim was positive for cocaine. A 28-years-old young man was under the effect of alfa-pyrroldino-pentiophenone at the time of the suicide, a psychostimulant with hallucinogenic effects [8].

The details of the 20 cases reported by the 17 articles reviewed are summarized in Table 1. They include sex and age of the 20 victims, their clinical and mental health history, the position of the body, veichle ignition status (on/off), the ligature material, anchor of the ligature, autopsy findings (external, internal and toxicological results), and cause of death assessed by the authors.

**Table 1:** Victims’ details (age, sex, clinical and mental health history), characteristics of the scenario (vehicle ignition status and body position), ligature materials, anchor of the ligature, autopsy findings (external, internal, and toxicological results), and the cause of death assessed by the authors

Cases no. - Articles	Sex/Age	Clinical and mental health history suicidal notes/attempts	Vehicle on/off	Position of the body	Ligature Material (S: Soft /H: Hard)	Anchor of the ligature	External findings	Internal findings	Toxicological findings	Cause of death assessed by authors
**1** - Hardwicke et al., 1985 [9]	M/67	depression over failing business and suicidal notes	On	Seated in the driver’s seat	H: hemp rope	Outside: a parked track close the vehicle	**Furrow**: recognizable, well-demarcated, oblique, incompletely encircling the neck from the laryngeal prominence to the left angle of the jaw. **Petechiae**: conjunctivae	**Neck muscles hemorrhages** **Petechiae**: subpleural **Other findings**: congestion of internal organs (lungs, liver and spleen).	-	Ligature strangulation
**2** - Durso et al., 1995 [10]	M/45	-	Off	Stretched in the front seats	H: rope	Inside: the car support handle of the front-door on passenger’s seat	**Furrow**: pale, poorly defined, oblique, incompleteley encircling the neck, superficial as it neared the nuchal region and devoid of bruises and abrasions.	**Other findings**: serohematic material from the respiratory orifices.	-	Hanging
**3** - Blanco Pampin et al., 2001 [11]	M/23	drug addiction, personality disorders and depression	Off	Seated in the rear seat	S: clothing belt	Inside: between the window glass and frame	**Furrow**: recognizable, well-demarcated, oblique incompletely encircling the neck. **Other findings**: hesitation marks at lower abdominal wall (*a knife was found near the body*).		cocaine and ethanol.	Hanging
**4** - Blanco Pampin et al., 2001 [11]	M/36	episode of auditory hallucinations	Off	Lying down in the rear seat	H: seat belt of the passanger’s rear seat	Inside: not specified	**Furrow**: recognizable, well-demarcated, completely encircling the neck.	**Neck soft tissues hemorrhages** **Fractures of thyroid cartilage** **Petechiae**: subpleural, epicardial	-	Hanging
**5** - Byard and James, 2001 [4]	M/47	depression and suicidal ideation	Off	Seated in front seat	H: seat belt	Inside: : not specified	**Furrow**: recognizable, well-demarcated with a weave pattern, incompletely encircling the neck. **Petechiae**: eyelids		-	Hanging
**6 -** Watanabe-Suzuki et al., 2002 [12]	M/20	-	On	Lying on the reclined driver’s seat	H: electrical cord	N.D.	**Furrow**: four ligature marks completely encircling the neck, pale, poorly defined, superficial and devoid of bruises and abrasions. **Petechiae**: conjunctivae	**Neck muscles hemorrhages** **Other findings**: hemorrhages of the left temporal muscle; subdural and subarachnoid hemorrhage. Collapse of both lungs with bullous emphysema	-	Suicidal or accidental (autoerotic) ligature strangulation
**7** - Zhao et al., 2008 [13]	M/59		On	Seated in the passenger’s front seat	H: hemp rope	Outside: a cherry tree	**Clear-cut decapitation injury** The head was found under the front seat **Petechiae**: conjunctivae	**Neck soft tissues/muscles hemorrhages** **Fractures of thyroid cartilage** **Cervical vertebrae fracture – dislocation** **Other findings**: brain swelling, lungs congestion	ethanol	Ligature strangulation with decapitation
**8** - Hejna et al., 2012 [14]	M/40	depression and suicidal notes	On	Seated in the driver’s seat	H: rope	Outside: a tree close to the vehicle	**Clear-cut decapitation injury** The head was found on the back seat	**Neck muscles hemorrhages** **Cervical vertebrae fracture – dislocation** **Other findings**: blood into airways and lungs	ethanol	Ligature strangulation with decapitation
**9** - Samberkar, 2012 [15]	M/63	-	On	Seated in driver’s seat	H: nylon rope	Outside: an iron gate	**Clear-cut decapitation injury** The head was between steering, dashboard and driver-seat door **Other findings: f**ace congestion and bitten tongue	**Neck muscles hemorrhages** **Cervical vertebrae fracture – dislocation**	-	Ligature strangulation with decapitation
**10** - Morild et al., 2012 [7]	M, -	-	On	Seated in the passanger’s front seat	H: nylon rope	Outside: a light post	**Clear-cut decapitation injury** with recognizable, and well-demarcated furrow. The head was found between the front seats **Furrow**: ligature mark near the severance plane	**Neck soft tissues/muscles hemorrhages** **Fractures of the hyoid bone and thyroid cartilage** **Fractures of the 3rd and 4th vertebrae**	-	Ligature strangulation with decapitation
**11** - Subirana-Domenech et al., 2014 [16]	M/51	-	On	Seated in the passenger’s front seat	H: rope	Outside: a bridge bannister	**Furrow**: recognizable, well-demarcated completely encircling the neck. **Petechiae**: eyelids and conjunctivae **Other findings**: bitten tongue, bloody mucus in nostrils and mouth.	**Neck muscles and thyroid cartilage hemorrhages** **Other findings**: section of thachea and partial rupture of the esophagus, laceration of both carotid arteries	-	Ligature strangulation
**12 -** Madea et al., 2015 [17]	M/67	-	Off	Seated in the passenger’s front seat	H: rope	Inside: the car support handle	**Furrow**: recognizable, well-demarcated, oblique, not completely encircling the neck. **Petechiae**: eyelids and conjunctivae	**Neck muscles and thyroid cartilage hemorrhages** **Other findings**: pulmonary and brain oedema.	-	Hanging
**13** - Madea et al., 2015 [17]	M/27	drug addiction and previous suicidal attempts	Off	Seated in the driver’s seat	H: plastic coated rope behind the headrest with a metal stick to twist the rope	Inside: the headrest	**Furrow**: recognizable, and well demarcated, only at the front of the neck **Petechiae**: conjunctivae.	**Neck muscles and thyroid cartilage hemorrhages**	ethanol	Ligature strangulation
**14** - Madea et al., 2015 [17]	M/48	-	On	Outside his car with driver’s door open	H: rope	Outside: a tree close to the vehicle	**Furrow**: recognizable, and well-demarcated, completely encircling the neck. **Petechiae**: eyelids and conjunctivae. **Other findings**: bitten tongue hemorrhages.	**Neck soft tissues/muscles hemorrhages** **Fractures of thyroid cartilage** **Other findings**: brain edema.	ethanol	Ligature strangulation
**15** Akcan et al., 2016 [18]	M/38	previous suicide ideation and suicidal notes	On	Seated in the passenger’s rear seat	H: rope	Outside: a tree close to the vehicle	**Furrow**: recognizable, well-demarcated, gently sloping upward toward the notch at left side of occipital region.	**Thyroid cartilage hemorrhges** **Other findings**: multiple wrist-cuts of on anterior side of both wrists.	Negative	Ligature strangulation
**16** - Barranco et al., 2018 [19]	F/48	major depression with previous suicide attempts	Off but rolling down the hill	Seated in the passanger’s rear seat	H: nylon rope	Outside: a wooden fence	**Furrow**: recognizable, well-demarcated, oblique, not completely encircling the neck.	**Larynx hemorrhages** **Petechiae**: subpleural petechiae.		Hanging
**17** - Chmura et al., 2018 [8]	M/28	depression with history of suicide attempts	Off	Outside his car with driver’s door open	First noose H: seat belt fastened Second noose S: shoelace	First Ligature: one end on the driver’s side pillar, the other end inserted into the standard buckle attached to the floor of the vehicle. Second Ligature: inside, to the steering wheel.	**First furrow**: recognizable, well demarcated, almost transversely around the neck. **Second furrow;** pale, poorly defined, oblique above the first one **Other findings**: bitten tongue hemorrhages.	**Neck soft tissues/muscles hemorrhages** **Fractures of thyroid cartilage** **Other findings**: epidermis abrasion on the posterior surface of hands and forearms and on the anterior surfaces of lower extremities; small amount of blood in the joint cavity; acute pulmonary emphysema.	alfa-pyrrolidino-pentiophenone	Hanging
**18** - Marchand et al., 2019 [20]	M/43	depression with suicide attempts	On	Seated in the driver’s seat	H: steel rope	Outside: a street light	**Clear-cut decapitation injury** The head was found on the passenger’s seat **Petechiae**: conjunctivae **Other findings**: face and nails cyanosis	**Neck soft tissues/muscles hemorrhages** **Fractures of the third vertebrae** **Fractures of thyroid cartilage** **Carotid arteries were sectioned** **Other findings**: pulmonary oedema with vascular congestion.	-	Ligature strangulation with decapitation
**19 -** Passos et al., 2023 [21]	M/49	depression and suicidal notes	On	Seated in the driver’s seat	H: rope	Outside: a metallic fixture on the wall	**Clear-cut decapitation injury** The head was found on the ground **Other findings**: hesitation wounds on left wrist	**Neck soft tissues/muscles hemorrhages** **Cervical vertebrae fracture – dislocation** **Fractures of the thyroid cartilage** (hyoid bone and thyroid gland were absent from the body)	antidepressants	Ligature strangulation with decapitation
**20 -** Lorenzoni et al., 2024 [22]	M/43	history of familiar and financial troubles	On	Seated in the driver’s seat	H: nylon rope	Outside: a tree close to the vehicle	**Clear-cut decapitation injury** The head was on the steering wheel **Furrow**: recognizable, and well-demarcated near the severance plane **Other findings**: face congestion	**Neck muscles, larynx, thyroid cartilage and aortic root hemorrhages** **Fractures of the 3rd and 4th vertebrae** Complete cross-section of the trachea and esophagus **Other findings**: pulmonary edema with a large amount of red blood cells	ethanol	Ligature strangulation with incomplete decapitation

In 19 out of 20 cases, hard ligatures were mostly used and represented by nylon, steel, plastic, or hemp materials, except in two cases where a clothing belt and shoelace were adopted. In 3 cases, a seatbelt was used as the hanging noose. Eight victims were seated in the driver’s seat, and other 9 cases in the passenger’s front or rear seats. The bodies of two victims were found outside the vehicle: one case was assessed as ligature strangulation, and the other as hanging with two nooses. In the latter case [8] the first noose was made from a seatbelt, and the second one was made from a shoelace, tightened between the deceased’s neck and the car’s steering wheel.

In all fatalities, the manner of death was assessed as suicide, except for the case #6 [12], where an accidental autoerotic death due to ligature strangulation was also considered, based on circumstantial data. No scratch marks are reported above or below the ligature marks, or other victims’ attempts to undo the noose.

The most common asphyxia death assessed was represented by ligature strangulation (13 out of 20 cases), followed by hanging (7 out of 20 cases). Based on the car ignition status, in 12 cases of ligature strangulation death occurred when the acceleration forces of the vehicle in motion were involved except for case #13 [17] occurred in a vehicle not running. According to the reconstruction of this suicide [17], the self-strangulation resembling a garroting was possible by carrying out by the victim’s own hand the stick used to twist the rope in a veichle not in motion. 8 victims in total were found in a motor vehicle off, not running because of it merely served as the place for the suicide among. All these cases were assessed as hanging except for the self-strangulation of case #13 [17]. In a single hanging death (case #16), the external compression to the neck was due to a moving car off rolling downward a hill [19]. In this case the kinetic energy causing the rope tight was represented by the gravitational force of the vehicle off on a slope.

In seven cases assessed as ligature strangulation, the decapitation also occurred with total separation of the head from the body due to the acceleration forces of the motor vehicle in motion. In one single death (case #20) assessed as ligature strangulation, an incomplete decapitation occurred [22]. In almost all cases, the head was found within the vehicle except in case #19 [21] where the head was found on the ground 10 mt behind the vehicle. In decapitation events resulting from vehicle-assisted ligature strangulation, a hard ligature was always stretched between the driver’s neck and stationary objects outside the vehicle, mostly represented by trees but also street light, light post, iron gate, and wall metallic fixture. Inside the vehicle the anchor of the ligature was not always reported but it was a stationary object represented by the car support handles or the headrest. No hanging case was related to decapitation.

Although hard ligature materials were mostly used, at external examination a recognizable, distinct, and well-demarcated furrow was observed in 13 cases only. In 3 deaths (cases #2, #6, #17) with a hard ligature material (i.e. electric cord, rope, cloting belt), the ligature mark was pale, poorly defined, superficial and devoid of bruises and abrasions. Most of the ligature marks were represented by a single furrow that encircled incompletely the neck except the seven cases with clear-cut decapitation. In two hanging cases (cases #4 and #17), and in three ligature strangulation (cases #6, #11, #14) the furrow encircled completely the neck. In case #17, a double furrow mark was found.

Other external signs commonly related to asphyxia deaths were represented by facial cyanosis and petechiae. Conjunctival or eyelids petechiae were reported in 9 out of 20 cases in total among which 7 ligature strangulation (with/without decapitation) and only 2 hanging. Bitten tongue hemorrhages were found in 3 cases of ligature strangulation (cases #9, #11, #14), and only one hanging (case #17). As internal findings, petechial hemorrhage spots on pleural or epicardial surfaces were only observed in 2 hanging cases and one ligature strangulation. Hemorrhages in the soft tissues or muscles of the neck were reported in 17 cases, mostly assessed as ligature strangulation, along with thyroid cartilage fractures in 7 victims (5 ligature strangulation and 2 hanging), and hemorrhages of the larynx and thyroid cartilage in 6 cases only (4 ligature strangulation and 2 hanging). Other internal findings commonly related to asphyxia deaths were represented by brain swelling and edema, lungs’ congestion and emphysema. No other signs often associated to hanging like the Amussat’s (transverse laceration of the intimal layer of carotid arteries), Friedberg’s (minute hemorrhages of the adventitia of the common carotid artery) or Brouardel’s signs (cervical prevertebral ecchymosis) were reported. In case #4 [11] hesitation marks at the lower abdominal wall were observed due to a previous suicide attempt. In all 20 cases reviewed, histological and/or immunohistochemical analyses were not performed on skin samples and/or soft tissues.

The distribution of the autopsy findings in the 20 cases are summarized in Table 2.

**Table 2:** The distribution of post-mortem findings reported in the 20 suicides by hanging and/or ligature strangulation inside a motor vehicle

Post-mortem finding *n* = 20	Hanging *n* = 7	Ligature strangulation *n* = 6	Ligature strangulation with decapitation *n* = 7
**Ligature materials**			
Seatbelt	3	0	0
Hemp rope	0	1	1
Rope not specified	2	3	2
Nylon rope	1	0	3
Plastic coated	0	1	0
Steel rope	0	0	1
Clothing Belt	1	0	0
Electrical cord	0	1	0
Shoelace	1	0	0
**External findings**			
Conjuntival and/or eyelids petechiae	2	5	2
Tongue hemorrhages	1	2	1
Facial congestion	0	1	3
Ligature mark poorly defined and pale	2	1	0
Well-demarcated ligature mark	6	5	2
**Internal findings**			
Neck muscles hemorrhages	2	5	7
Hemorrhages of the thyroid cartilage and larynx	2	3	1
Fractures of the thyroid cartilage	2	1	4
Fractures of the hyoid bone	0	0	1
Cervical vertebrae fracture-dislocation	0	0	7
Brain edema	1	1	1
Pulmonary congestion and emphysema	1	2	3
Blood into airways and lungs	1	1	1
Visceral petechiae	2	1	0

In all decapitation cases due to ligature strangulation, the injuries showed a common morphology: a clear-cut decapitation wound with hemorrhagic infiltration of edges and peripheral abrasion along with fracture-dislocation of the upper cervical vertebrae. The severance plane was mostly located at the level of the larynx with fractures of the thyroid cartilage (cases #7, #10, #18) or the hyoid bone (case #10). Fractures of the third and the fourth cervical vertebra were reported in 3 cases (cases #10, #18, #20). No fractures of cervical vertebrae were related to hanging.

Differentiating between hanging and ligature strangulation in cases of stationary vehicle suicides (where the vehicle’s force isn’t acting as an external force) was not always easy for the Authors of the articles revised. In 2 out of 7 hanging cases, the prevalent mechanism of death was related to vascular occlusion of the main arteries and veins of the neck. In the other 2 cases assessed as hanging (cases #3 and #5), it is worth of mentioning the lack of internal signs of asphyxia like soft tissue/muscles hemorrhages, pulmonary edema, and visceral congestion, except for the external distinct ligature marks. An additional case of ligature strangulation inside a vehicle stopped has been observed by the Authors and it is briefly reported below.

## Case report

**Fig. 2:**
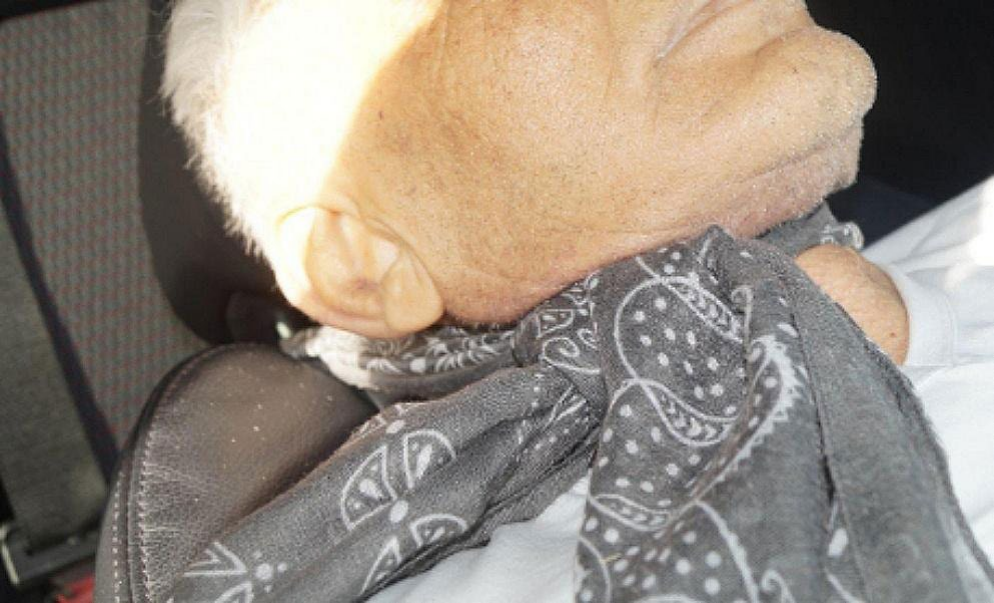
The pashmina scarf wrapped around the neck and the headrest at the driver’s seat

A 65-years-old white male was found dead inside his car parked at a gas station. The car doors were closed and locked, with the front right window glass broken by the emergency medical service personnel. The driver backrest was almost fully reclined. The man’s body was found lying on it in a supine position. A ‘pashmina’ scarf was wrapped around both his neck and the headrest. The scarf was knotted at the front right side of the neck (Fig. 2). The lower left limb was positioned with the thigh flexed on the abdomen, the knee slightly flexed and the left foot’s plantar surface pushing against the dashboard.

The pants were unfastened, and the left hand was just on the trouser zip open. A half-empty bottle of whiskey and a plastic bottle containing a green-yellow liquid that smelled like gasoline were found. Surveillance cameras recorded the vehicle in the parking area all the time, and nobody else except the victim was seen inside the car.

**Fig. 3:**
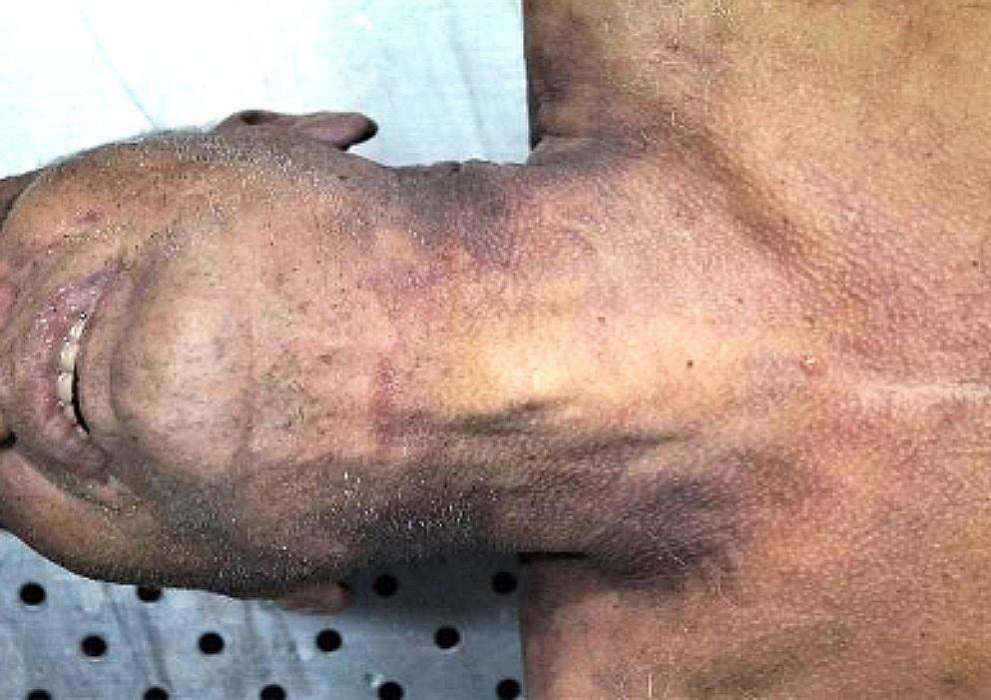
Anterior cervical region with incomplete furrow and faint reddish discolored skin

At external examination, the furrow showed a pale-yellow parchment-like appearance that did not completely encircle the neck. Above the furrow, some faint reddish discolored skin was observed (Fig. 3).

Conjunctival pinpoint hemorrhages were present at the right eye only. At internal examination, no macroscopic or microscopic hemorrhages were observed within the neck muscles or in close proximity of the thyroid gland, hyoid bone, or thyroid cartilage. The cervical spine, the hyoid bone and the thyroid cartilage were intact. The lungs displayed no signs commonly related to asphyxia like overinflation, pleural or subepicardial petechiae, or acute emphysema, except for brain swelling and visceral congestion. Toxicological analysis, performed on blood samples by gas chromatography–mass spectrometry (GC-MS), showed 1.1 g/L of ethyl alcohol.

Ligature strangulation was assessed as cause of death. Although in the beginning an accidental autoerotic death was also considered, the manner of death was finally assessed as suicide according to circumstantial information coming from the examination of nearby closed-circuit television (CCTV) footage and the discovery of suicidal notes inside the vehicle. The victim was stressed about his familial condition; after recently getting divorced, he started a new relationship with a divorced woman who was not accepted by his family. Reports from the woman’s relatives mentioned his usual tendency to get angry easily and episodes of domestic violence.

## Discussion

In the literature, a few suicides by ligature strangulation and/or hanging inside a car have been reported. Vehicles are generally unsuitable for hanging or ligature strangulation because they are narrow in space. There is no room enough to suspend the body from a rope or to secure the rope to a rigid support [23]. All the hanging deaths reported in the literature review occurred in vehicles off, and none were related to decapitation. This is surprising since in judicial hangings, death is commonly caused by fracture-dislocation of the upper cervical vertebrae with transection of the cord, and decapitation can occur if the victim falls too far from the gallows [24]. If the victim falls an insufficient distance, strangulation can occur rather than breaking the neck. Instead, this comprehensive review shows that decapitation occurred only in 7 cases but classified as ligature strangulation due to the acceleration forces of the vehicles in motion. In ligature strangulation, the forward motion of the vehicle must be considered the external physical force acting on the neck through a tightened ligature [16, 25]. In hanging, the victim’s own body weight exerts compressive forces on the neck by a ligature, such as the vehicle’s seat belt, a rope, or clothing belt. All hanging cases are reported as atypical, unusual, and incomplete based on the body position and the configuration of the ligature marks. All ligature marks were represented by a single oblique furrow that encircled incompletely the neck, except for the seven cases with clear-cut decapitation. In two hanging (cases #4 and #17), and in three ligature strangulation (cases #6, #11, #14) the furrow completely encircled the neck. In the other three ligature strangulation, the furrow was incompletely encircling the neck (cases #1, #13, #15), as well as in 5 hanging cases where the ligature mark was oblique and superficial as near the knot (cases #2, #3, #5, #12, #16).

In ligature strangulation, decapitation requires a fast acceleration of the vehicle in motion and the use of an inelastic ligature [26], such as a cable steel or a nylon twisted rope. Vehicle-assisted self-strangulations can lead to decapitation when a ligature is pulled between the victim’s neck and a stationary object outside the car as the anchor of the ligature and the driver starts the vehicle [20]. These cases remain relatively uncommon and require careful investigation and analysis of the death scene to rule out other causes and manner of death. In cases where the vehicle is off and its force isn’t acting as an external force on the neck, differentiating between hanging and ligature strangulation can be a complex task because of the challenge in identifying the underlying mechanism of death (airway obstruction, vascular occlusion of the main arteries and veins, and carotid sinus reflex). This is especially true in cases where no common signs of asphyxia are found at external and internal examination such as in the additional case study reported by the Authors.

In the forensic literature, the classification of asphyxia is not uniform between authors, and similar cases can be assessed differently by forensic pathologists. According to a classification of asphyxia proposed by Sauvageau and Boghossian [27, 28], three different types of strangulation based on the source of the external pressure on the neck can be determined: hanging involves a constricting band tightened by the body’s gravitational force; ligature strangulation involves a force other than the body weight contributing to asphyxiation; manual strangulation entails pressure applied to the neck by hands, forearms, or other limbs. Nevertheless, the authors recommend labeling all asphyxia deaths from external compression to the neck as strangulation. If a specific subtype (manual, ligature, or hanging) cannot be determined, it should be classified as ‘strangulation nos’ (not otherwise specified) [27].

In a recent review, the definition of suicidal ligature strangulation has been expanded to include cases involving the attachment of the ligature to additional weights or devices to sustain the compression to the neck [29]. This recommendation can be applied to the self-garroting case #13, where the victim used a metal stick to tighten the rope around the neck, and to the additional case example observed by the Authors. In our case, no signs of asphyxia were found, except for a broad pale furrow due to soft noose and some conjunctival petechiae at the right eye only. No other hemorrhages were present at external or internal examination. This is not surprising. It is known that in asphyxia victims due to compression of the neck using a ligature postmortem findings on external and internal examination may vary considerably depending on the type of violent neck trauma, the intensity with which a victim resisted, as well as the intensity and duration of neck compression [25].

Some authors [24] report that in more than half of the hanging cases, there are no injuries on internal examination of the neck structures and hemorrhages in the neck muscles are often absent [25]. The likelihood of fractures in the laryngeal and hyoid structures varies according to the degree of ossification and depending on age [25]. At external examination, a recognizable, distinct, and well-demarcated furrow was described only in 13 out of 20 cases in total. In the other 3 cases (2 hanging and 1 ligature strangulation), the furrow was poorly defined and pale, devoid of bruises and abrasions at the upper or lower margins, although the ligatures were mostly hard (i.e., electrical cord, rope) except in case #17, in which a shoelace was used [8]. These findings can exhibit significant variability, predominantly influenced by the material used as ligature, its characteristics, and texture. If the ligature is a soft material, such as a towel, the groove might be faint and pale, indistinct, barely visible, poorly defined [30].

In a comparison of post-mortem findings observed in homicidal and suicidal ligature strangulation [31], bleedings in the neck muscles seldom occurred in suicides. However, the laryngo-hyoid injuries could be helpful in the differentiating suicides from homicides if more than a single thyroid horn fracture or a laryngeal soft tissue trauma is present. In a revision of 116 cases, of suicidal ligature strangulation the number of laryngo-hyoid fractures and hemorrhages was generally low and extremely uncommon [32], probably due to the severe compression of the blood vessels brought about by strangulation [17].

In this comprehensive review, facial congestion and petechiae (conjunctival or at eyelids) were reported only in 9 out of 20 cases in total, among which 7 out of 11 cases of ligature strangulation (with/without decapitation) and only 2 hanging. According to Di Maio and Di Maio [24] petechiae of the conjunctivae can be mostly observed in ligature strangulation cases compared to hanging cases, because, unlike in hanging, there is no complete occlusion of the vessels and blood continues to go into the head from the vertebral arteries. Tongue hemorrhages were also mostly observed in ligature strangulation (3 cases) and in one hanging victim. In ligature strangulation, hemorrhages can mostly occur at the tongue due to the anatomical vicinity of the jugular and carotid vessels and congestion of the face and neck with increased intravascular cephalic venous pressure [33]. Hemorrhages in the soft tissues and muscles of the neck were again found mostly in ligature strangulation with decapitation (7 cases), along with thyroid cartilage fractures and one fracture of the hyoid bone.

Unfortunately, no internal signs often associated to hanging like the Amussat’s, Friedberg’s, or Brouardel’s signs, were reported. Petechial hemorrhages spots on pleural or epicardial surfaces were only reported in 2 cases of hanging and one ligature strangulation.

In two hanging cases and in the additional case reported by the Authors, no internal findings of asphyxia were found. The lack of internal sings of asphyxia can be explained in these cases by the combination of the three main mechanisms involved in death following the compression to the neck (airway obstruction, vascular occlusion of main arteries and veins, carotid sinus reflex). These mechanisms can act independently or in combination. For example, when the role of the carotid sinus reflex is prevalent, both external and internal findings in the form of local hemorrhages may be easily absent due to the rapid death. The vagal reflex mechanism can be a poor contributing factor in the case of hanging, as cerebral ischemia is primarily caused by the compression of the arteries with no distinct related injuries or hemorrhages of the neck.

Indeed, determining the type of asphyxia death (ligature strangulation or hanging) for victims who died due to compression to the neck by a ligature can be challenging [34, 35]. This is also due to the misconception that strong pressure is needed on the neck to occlude the airways and the arterial vessels of the neck. It has been demonstrated that a force of 8–12 kg is necessary to occlude the airways [36], whereas a lower amount of pressure is enough to occlude the carotid arteries and veins but not the vertebral arteries that need a load of 30 kg approximately [37].

Therefore, the weight of the head against a noose can be sufficient to occlude the carotid arteries and cause death [24]. In this context, the carotid sinus reflex can be triggered both by a minimal pressure to the neck, at the level of carotid artery bifurcation, or by longitudinal stretching of the carotid artery [36]. The hypothesis of manual compression triggering the carotid sinus reflex and fatal cardiac arrhythmias has been also suggested as a mechanism of death [38]. An individual with minimal autoerotic experience by ligature may unintentionally suffer immediate syncope or coma by carotid sinus pressure [36]. The hypothesis of an accidental autoerotic death was raised by the Authors in case #6 [12] and considered in our case report due to the left hand found close to the paint zip open. This hypothesis was discarded soon after the discovery of a suicidal note inside the car.

The accidental autoerotic death by ligature is commonly the consequence of the failure of a release mechanism after using hypoxia to enhance the sexual response while masturbating [39, 40]. The individuals choose hanging or ligature strangulation to obtain compression of the carotid arteries and stimulation of the carotid sinus reflex to produce an orgasm-enhancing effect. Usually, autoerotic asphyxia is performed in indoor settings (victim’s own home) [36] but, sometimes, bodies could be found in public areas (open fields but also cars) [41]. The exposure of the genitals, a complete or partial nudity, the presence of pornographic material on the scene, and the victim’s hands near the genitals could suggest antemortem sexual activity [42, 43]. The presence of semen does not necessarily indicate evidence of antemortem masturbation since ejaculation may occur because of neurophysiological reflexes [44, 45].

Therefore, the discrepancies between external and internal findings and the suicidal or accidental asphyxia deaths by ligature strangulation and/or hanging are not surprising. A comparative study of the pathological features found in ligature strangulation, suicidal hanging, and autoerotic deaths shows that internal injuries are uncommon in suicidal hanging and in autoerotic asphyxia [36]. In both cases, the severe compression of the blood vessels as well as the carotid sinus reflex can easily determine the absence of hemorrhages at the neck muscles and even around laryngeal and hyoid fractures. Although recommended by several Authors for the assessment of the vitality, immunohistochemical analysis of the ligature mark has not found yet general agreement among the scientific community. Validation protocols to promote consistency and comparability among immunohistochemical markers and to improve their reliability in routine diagnostics of vital reaction are still needed [30, 46–48].

The transfer of information collected at the death scene among investigators and the forensic pathologists is crucial for the reconstruction of events preceding the death and to assess the correct manner of death. In our case, the homicidal hypothesis was excluded based on the record of the video surveillance camera available at parking area.

## Conclusions

This comprehensive review has summarized the autopsy findings from 20 cases of suicidal asphyxia deaths by ligature strangulation and hanging inside motor vehicle off and in motion. These cases are rare and sometimes challenging for forensic pathologists. The manner of death cannot always be clear and evident. The data provided can be useful for the comparison in asphyxia deaths from external compression to the neck. If a specific subtype (manual, ligature, or hanging) cannot be determined, the case under investigation should be classified as a strangulation not otherwise specified according to Savageau and Bogossian [27].

## Data Availability

The data presented in this study are available on request from the corresponding author. The data are not publicly available due to privacy restriction.
